# The effect of cancer exemption in mandatory-access prescription drug monitoring programs among oncologists

**DOI:** 10.1093/jncics/pkad006

**Published:** 2023-02-08

**Authors:** Ilana Graetz, Xin Hu, Xu Ji, Martha Wetzel, Courtney R Yarbrough

**Affiliations:** Department of Health Policy and Management, Emory University Rollins School of Public Health, Atlanta, GA, USA; Department of Health Policy and Management, Emory University Rollins School of Public Health, Atlanta, GA, USA; Department of Pediatrics, Emory University School of Medicine, Atlanta, GA, USA; Aflac Cancer and Blood Disorders Center, Children's Healthcare of Atlanta, Atlanta, GA, USA; Department of Health Policy and Management, Emory University Rollins School of Public Health, Atlanta, GA, USA; Department of Health Policy and Management, Emory University Rollins School of Public Health, Atlanta, GA, USA

## Abstract

To address the opioid epidemic, some states mandate that prescribers review a state-run prescription drug monitoring program (PDMP) database before prescribing opioids. We used Medicare Part D prescriber data from 2013 (baseline) to 2019 to examine the association between state mandatory-access PDMPs, with and without a cancer exemption, and changes in the percent of oncologists’ patients with any opioid fill per year, stratified by oncologists’ baseline prescribing volume. Among 9746 medical or hematologic oncologists, the proportion of patients prescribed opioids declined after states implemented mandatory-access PDMPs without a cancer exemption overall (−0.49 percentage point, 95% confidence interval = −0.78 to −0.20 percentage point) and among those with above-median baseline prescribing, but not in states with a cancer exemption (−0.16 percentage point, 95% confidence interval = −0.50 to 0.18 percentage point) or with below-median baseline prescribing. Carefully designed mandatory-access PDMPs with cancer exemptions minimize unnecessary reductions in prescription opioid treatments among oncology patients in need of pain management.

In response to the ongoing opioid epidemic (1-6), most states enacted prescription drug monitoring programs (PDMPs), which are state-run databases of patients’ controlled substances prescribing histories (7). Increasingly, states mandate that providers consult the PDMP before prescribing opioids. Some states exempt prescribers from these requirements for their patients with cancer. As mandatary-access PDMP policies expand, there is a need for research on effective policies that curb overprescribing while limiting unintended consequences among patients with appropriate indications for opioids, such as those undergoing cancer treatment.

Our prior study using data through 2017 found similar reductions in opioid prescribing by oncologists in states with a mandatory-access PDMP with or without a cancer exemption (8). Since 2017, the number of states with a cancer exemption increased from only 3 states to 12 states by 2019 (Supplementary Table 1, available online). With more state variation in PDMP policies and 2 additional years of data, we updated our analyses. Furthermore, because the impact of the policy may be different for higher- vs lower-volume prescribers (9), we also conducted stratified analyses by baseline quartile of opioid prescribing volume.

In this cohort study using physician-level Medicare Part D Prescriber files for 2013 to 2019 (10), we evaluated changes in opioid prescribing associated with mandatory-access PDMP policies among physicians specialized in medical or hematologic oncology and included in the 2013 data with at least 1 follow-up year in the 2014-2019 data. We used the baseline 2013 records to categorize oncologists into quartiles based on the proportion of their Part D patients with any opioid prescription fills. We then assessed the proportion of oncologists’ patients covered by Medicare Part D to whom they prescribed any opioids each year from 2014 to 2019. States were categorized as having a mandatory-access PDMP if they required prescribers to review the PDMP database before writing an initial opioid prescription for a patient. For each year, we classified prescribers based on their state policy that year as having no mandatory-access PDMP or a mandatory-access PDMP with or without a cancer exemption (Supplementary Table 1, available online).

We used a difference-in-difference model (11) with physician and year fixed effects to measure changes in prescribing overall and stratified by baseline quartile opioid prescribing volume. This approach measured within-physician changes in annual opioid prescribing after the implementation of a mandatory-access PDMP with and without cancer exemption compared with physicians in states with no mandates. The nonstratified model included a year and quartile interaction term to allow for differential time trends by baseline opioid prescribing volume. The postestimation “margins” command was used to generate average predicted probabilities by PDMP status. Because values are suppressed in the data for physicians with fewer than 11 patients taking opioids, we conducted multiple imputations with an interval regression imputation approach (12). Analyses with and without imputations showed similar results. Statistical significance is determined by *P* less than .05 using 2-sided tests. Because this study used publicly available data, institutional review board approval and informed consent were waived.

Baseline characteristics of the 9749 included oncologists are shown in Supplementary Table 2 (available online). Adjusted results show that 20.56% of Part D patients filled opioid prescriptions by oncologists in states without a mandatory-access PDMP compared with 20.07% in states after they implemented a mandatory-access PDMP without a cancer exemption for oncologists (−0.49 percentage point [ppt], 95% confidence interval [CI] = −0.78 to −0.20 ppt) and 20.40% in states after they implemented a mandatory-access PDMP with a cancer exemption (−0.16 ppt, 95% CI = −0.50 to 0.18 ppt; Figures 1 and 2). In stratified analyses, oncologists with above-median baseline prescribing experienced statistically significant reductions in opioid prescribing after their states implemented a mandatory-access PDMP without a cancer exemption (Quartile 4: −0.85 ppt, 95% CI = −1.56 to −0.15 ppt; Quartile 3: −0.48 ppt, 95% CI = −0.95 to −0.01 ppt). Among below-median baseline prescribers and oncologists in states where the mandatory-access PDMP had a cancer exemption, the reduction in opioid prescribing was smaller and statistically nonsignificant (Figure 2).

**Figure 1.**
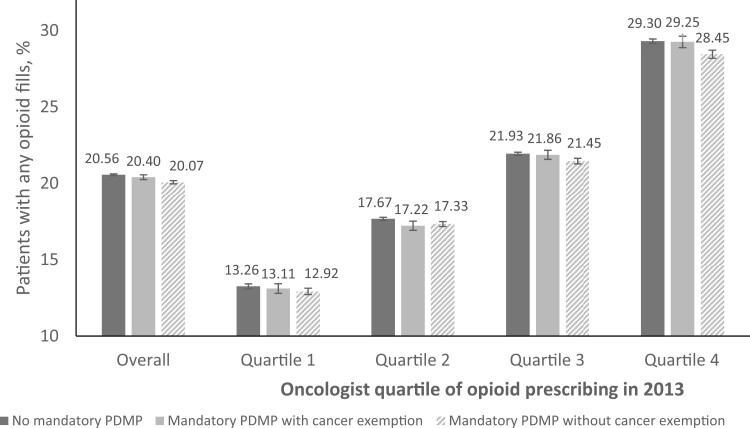
Adjusted percent of patients treated by a medical or hematologic oncologist with any opioid prescription, by oncologists’ quartile of opioid prescribing in 2013 (ie, baseline). Adjusted changes calculated using a difference-in-difference model with physician and year fixed effects to measure yearly changes in the proportion of patients with any opioid prescription overall and stratified by oncologists’ baseline quartile of prescribing. Postestimation “margins” command was used to generate average predicted probabilities for no mandatory-access states and mandatory-access states with and without cancer exemption. No mandatory prescription drug monitoring program (PDMP) includes observations of oncologists practicing in 1 of 13 states that did not implement a mandatory-access PDMP before 2019 and observations of oncologists practicing in states that implemented a mandatory-access PDMP between 2014 and 2019 during the years before their state implemented the policy.

**Figure 2.**
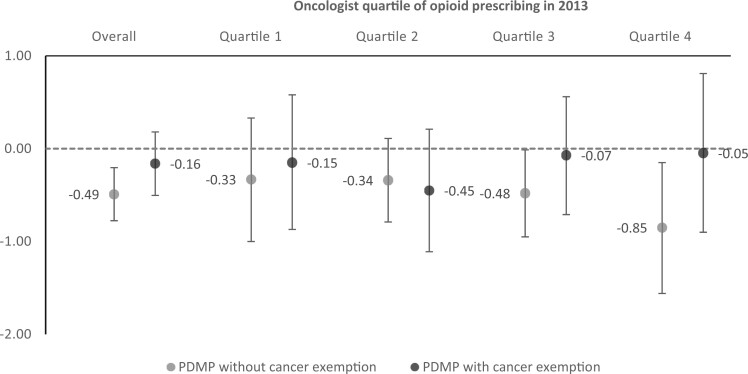
Adjusted changes in the proportion of cancer patients with any opioid prescription associated with the implementation of mandatory-access prescription drug monitoring program (PDMP) with and without a cancer exemption, by oncologists’ baseline quartile of opioid prescribing. Adjusted changes calculated using a difference-in-difference model with physician and year fixed effects to measure changes in the proportion with any opioid prescription overall and stratified by baseline quartile of prescribing. Overall model includes a year and quartile interaction term to allow for a differential time trend by baseline opioid prescribing volume. No mandatory PDMP includes oncologists practicing in 1 of 13 states that did not implement a mandatory-access PDMP before 2019 and oncologists practicing in states that implemented a mandatory-access PDMP between 2014 and 2019 during the years before their state implemented the policy.

Our study found that among oncologists with the highest initial opioid prescribing, the proportion of their patients that filled opioid prescriptions opioids declined by 2.90% (−0.85 ppt/29.3 ppt) in states with mandatory-access PDMPs without an exemption for cancer compared with statistically nonsignificant decline of 0.17% (−0.05 ppt/29.3 ppt) in states with an exemption. Mandatory-access PDMPs are intended to encourage more appropriate opioid prescribing by providing information to help identify patients who may be “doctor shopping” or have a substance use disorder (13). However, a prior analysis found that only one-third of the decline in opioid prescribing was due to the information provided by the database, and more than two-thirds of the decline was attributable to the additional hassle of mandatory record checks (14). Accumulating evidence finds that federal and state efforts to restrict opioid prescribing had unintended consequences for patients explicitly excluded from the 2016 Centers for Disease Control and Prevention guidelines or with appropriate indications for opioid analgesics (8,15,16). Patients with cancer are particularly vulnerable because they frequently experience undertreated pain (17). Amid recent efforts to implement mandatory-access PDMPs with specific provisions to avoid unintended consequences for patients requiring opioid analgesia, our study adds new evidence that mandatory-access PDMPs with an exemption for cancer shield patients with cancer from unnecessary reductions in opioid access.

Our analyses had limitations. First, we lacked patient-level information, including cancer diagnosis and prognosis that may affect the clinical indication for opioid prescription. However, because mandatory-access PDMPs are intended to change prescriber behavior, our difference-in-difference analyses controlled for time-stable provider characteristics and measured only within-provider changes associated with mandatory-access PDMPs. Second, our finding was limited to medical or hematologic oncologists’ prescription patterns for Medicare Part D beneficiaries, which may not generalize to other specialists, prescribers such as nurse practitioners, or patients.

In this cohort study of 9746 oncologists, we found reductions in the proportion of their patients prescribed opioids after mandatory-access PDMPs were enacted. However, among prescribers in states that implemented mandatory-access PDMPs with a cancer exemption, these reductions were small and not statistically significantly different than in states without a mandatory-access PDMP. Carefully designed mandatory-access PDMP policies with an exclusion for cancer minimize unnecessary reductions in accessing prescription opioid treatments among patients with cancer who have appropriate indications for opioids.

## Supplementary Material

pkad006_Supplementary_Data

## Data Availability

The data underlying this article are available from the Centers for Medicare and Medicaid Services at https://data.cms.gov/provider-summary-by-type-of-service/medicare-part-d-prescribers.
