# Supplementary material for: The effect of cancer exemption in mandatory-access prescription drug monitoring programs among oncologists
Source: JNCI Cancer Spectr. 2023 Feb 8;7(2):pkad006. doi: 10.1093/jncics/pkad006 (PMC9978313; doi:10.1093/jncics/pkad006)
Supplement: pkad006_Supplementary_Data [file pkad006_supplementary_data.pdf]

Supplementary Materials

Supplementary Table 1. Year Mandatory-Access PDMP Implemented by State and Cancer Exemption from 2012 to 2019

Supplementary Table 2. Descriptive Characteristics of Medical or Hematologic Oncologist Who Prescribed Opioids in 2013 By Quartile of Opioid Prescribing with Imputation for Suppressed Values

Supplementary Table 1. Year Mandatory-Access PDMP Implemented by State and Cancer Exemption from 2012 to 2019

State	Year Mandatory-Access PDMP Implemented	Exemption for patients with cancer
Alabama		
Alaska	2017	
Arizona	2017	Yes
Arkansas	2017	
California	2018	
Colorado	2018	Yes
Connecticut	2015	
Delaware		
District of Columbia		
Florida	2018	
Georgia	2018	Yes
Hawaii	2018	
Idaho		
Illinois	2018	Yes
Indiana	2021	
Iowa	2018	
Kansas		
Kentucky	2012	
Louisiana	2018	Yes
Maine	2017	
Maryland	2018	Yes
Massachusetts	2016	
Michigan	2018	
Minnesota		
Mississippi	2018	Yes
Missouri		
Montana		
Nebraska		
Nevada	2018	
New Hampshire	2017	
New Jersey	2015	
New Mexico	2017	
New York	2013	
North Carolina		
North Dakota	2018	
Ohio	2015	Yes
Oklahoma	2015	
Oregon		
Pennsylvania	2017	
Rhode Island	2016	
South Carolina	2017	
South Dakota		
Tennessee	2013	
Texas	2019	Yes
Utah	2018	
Vermont	2013	Yes
Virginia	2017	
Washington	2019	Yes
West Virginia	2013	Yes
Wisconsin	2017	
Wyoming		

Note: States categorized as having a mandatory-access PDMP if they required prescribers to review the PDMP database before writing an initial opioid prescription for a patient and again at regular intervals

Supplementary Table 2. Descriptive Characteristics of Medical or Hematologic Oncologist Who Prescribed Opioids in 2013 By Quartile of Opioid Prescribing with Imputation for Suppressed Values

	Total		Lowest Volume Quartile 1		Quartile 2		Quartile 3		Highest Volume Quartile 4	
	**N**	**Col %**	**N**	**Col %**	**N**	**Col %**	**N**	**Col %**	**N**	**Col %**
	**9749**		2439		2436		2447		2427	
Gender: Female	2884	29.6%	952	39.0%	696	28.6%	574	23.5%	662	27.3%
Region										
Midwest	2190	22.5%	523	21.4%	589	24.2%	556	22.7%	522	21.5%
Northeast	2327	23.9%	835	34.2%	589	24.2%	498	20.4%	405	16.7%
South	3373	34.6%	549	22.5%	766	31.4%	922	37.7%	1,136	46.8%
West	1859	19.1%	532	21.8%	492	20.2%	471	19.2%	364	15.0%
PDMP status in 2013										
No PDMP	8520	87.4%	2004	82.2%	2165	88.9%	2185	89.3%	2166	89.2%
PDMP without cancer exemption	1155	11.8%	424	17.4%	251	10.3%	240	9.8%	240	9.9%
PDMP with cancer exemption	74	0.8%	11	0.5%	20	0.8%	22	0.9%	21	0.9%

Note: Because values are suppressed in the data for physicians with fewer than 11 patients taking opioids, we conducted multiple imputations with an interval regression imputation approach.
